# Heart-targeted amelioration of sepsis-induced myocardial dysfunction by microenvironment responsive nitric oxide nanogenerators in situ

**DOI:** 10.1186/s12951-022-01457-y

**Published:** 2022-06-07

**Authors:** Minzhi Ouyang, Xiangnan Ouyang, Zefang Peng, Minghui Liu, Ganqiong Xu, Zhen Zou, Ming Zhang, Quanliang Shang

**Affiliations:** 1 Department of Ultrasound Diagnosis, Second Xiangya Hospital, Central South University, Changsha, 410011 China; 2 Department of Ultrasound, Hunan Provincial People’s Hospital, The First Affiliated of Hunan Normal University, No. 61 Jiefang Road (W), Changsha, China; 3 Laboratory of Chemical Biology and Traditional Chinese Medicine Research, Ministry of Education, College of Chemistry and Chemical Engineering, Hunan Normal University, Changsha, 410081 China

**Keywords:** Sepsis, Targeted delivery, Microenvironment change, Gas therapy, Cardiac dysfunction alleviation

## Abstract

**Background:**

A balanced endogenous level of bioavailable nitric oxide (NO) plays a key role in maintaining cardiovascular homeostasis. The bioactive NO level in the cardiomyocytes was much reduced during sepsis. However, it is clinically challenging for the NO gas therapy due to the lack of spatial and temporal release system with precise control. The purpose of this study is to design a NO-releasing biomaterial with heart-targeted capability responsive to the infectious microenvironment, thus ameliorating lipopolysaccharide (LPS)-induced cardiac dysfunction.

**Results:**

The heart-targeted NO delivery and in situ releasing system, PCM-MSN@LA, was synthesized using hollow mesoporous silica nanoparticles (MSN) as the carrier, and L-arginine (LA) as the NO donor. The myocardial delivery was successfully directed to heart by specific peptide (PCM) combined with low-intensity focused ultrasound (LIFU) guidance. The myocardial system synthesized NO from the LA released from PCM-MSN@LA in the presence of increased endogenous nitric oxide synthase (NOS) activity induced by LPS. This targeted NO release in situ achieved extraordinary protective effects against LPS-challenged myocardial injury by reducing the recruitment of inflammatory cells, inhibiting oxidative stress and maintaining the mitochondria integrity. In particular, this protection was not compromised by simultaneous circulation collapse as an adverse event in the context.

**Conclusions:**

PCM-MSN@LA + LIFU exhibited extraordinary cardioprotective effects against severe sepsis in the hearts of LPS-treated animals without the side effect of NO diffusion. This technology has great potential to be served as a novel therapeutic strategy for sepsis-induced myocardial injury.

**Supplementary Information:**

The online version contains supplementary material available at 10.1186/s12951-022-01457-y.

## Background

Sepsis is caused by the dysregulated host response to infection accompanied by life-threatening organ dysfunction. The mortality of sepsis in 2017 was approximately 11.0 million representing 19.7% of deaths worldwide [1, 2]. Lately, the COVID-19 pandemic has been recognized as a public health emergency of global concern with sepsis as an overlooked killer in COVID-19 patients [3]. Septic cardiomyopathy is a leading type of sepsis-associated organ injury and plays a central role in increasing mortality. For instance, the mortality risk exceeded 70–90% in patients who had developed heart failure in sepsis [4–6]. The mechanisms involved in septic cardiomyopathy include dysregulated inflammatory response, oxidative stress and mitochondrial damage [7]. Although advances in understanding the mechanism have been achieved, drug-resistant bacterial mutants and the complicated pathophysiology have hampered the clinical outcomes of septic cardiomyopathy. Reprogramming of the septic heart microenvironment has become an indispensable supportive treatment for relieving heart failure by maintaining cardiac homeostasis in the context of sepsis.

Nitric oxide (NO) is an important signalling molecule in cardiovascular diseases. NO donors successfully ameliorated the impaired myocardial microenvironment by inhibiting inflammatory cell recruitment, preventing excessive oxidant responses and stabilizing mitochondrial homeostasis [8–10]. Following the reactions of peroxynitrite (O^2−^ + NO → ONOO^−^), elevated reactive oxygen species (ROS) in sepsis could consume NO, consequently abrogating NO bioavailability and deteriorating organ dysfunction [11, 12]. Thus, increasing the NO content at the early stage of sepsis has the potential to maintain NO activity and further reduce ROS production without the risk of producing excessive peroxynitrite (ONOO^−^). However, the delivery and employment of NO to the myocardium have remained challenging, mainly due to the random diffusion and reactive chemical nature of NO in the circulation. Besides, taking long-term organic nitrates led to cyanide accumulation in the body [13]. As an alternative, NO-donating nanoparticles have attracted extensive attention due to their stability in circulation enabling modification by a variety of substances. To ensure the therapeutic effects of NO, the nanoparticles should target the myocardium precisely followed by a stimuli-responsive NO release.

MSNs are used in this study for LA conjugation to enhance the stability of the NO donor. Attaching specific moieties such as peptides to the surface of MSN@LA nanoparticles could provide robust targeted delivery capability [14]. PCM, a 20-mer peptide (WLSEAGPVVTVRALRGTGSW) isolated by phage display, could bind to primary cardiomyocytes with a 180-fold higher efficiency than the control, at the same time showing great specificity [15]. Therapeutic ultrasound, known as low-intensity focused ultrasound (LIFU), has been recommended as an efficient approach to further improve targeted delivery to the myocardium [16, 17]. When exposed to the LIFU irradiation, the uptake of bioactive agents was enhanced in specific tissues due to a transient increase in the cell membrane permeability [18, 19]. Therapeutic ultrasound usually has a frequency range of 0.75–3 Hz, within which the frequency of 1–2 Hz has been mostly used [19]. Therefore, the dual system employing the PCM-mediated targeting and LIFU irradiation is promising to precisely transfer LA to the myocardium.

**Fig. 1:**
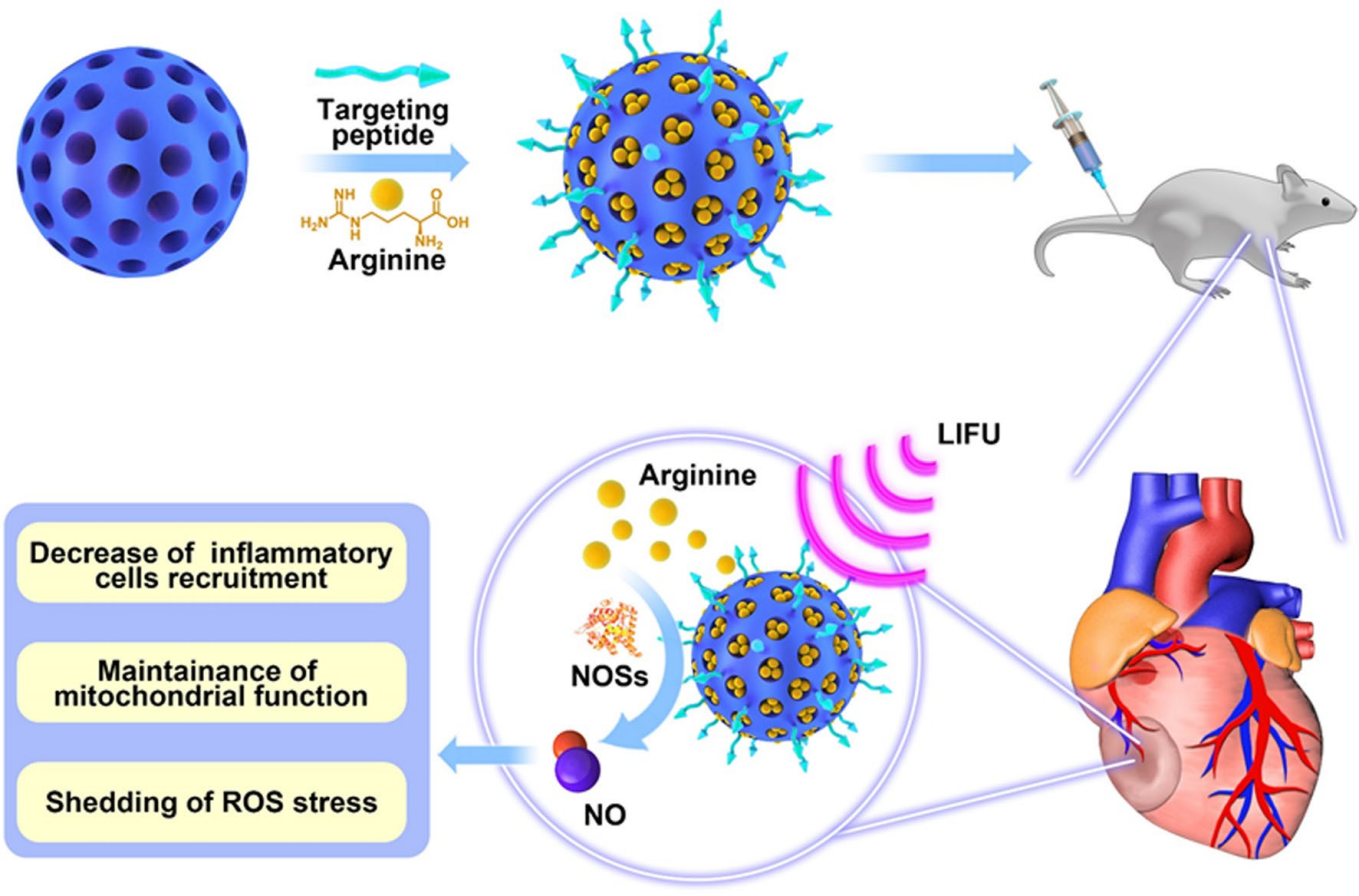
NO liberation in septic heart from PCM-carried and L-arginine loaded porous MSN, named PCM-MSN@LA, combined with the LIFU, preventing the myocardial dysfunction

Various polymer- and lipid-based NO donor carriers have shown some benefits in promoting vasodilation in stroke [20], restoring glucose homeostasis [21] or treating cancer [22, 23], yet not related to septic cardiomyopathy management. As known, the NO serves as a cellular messenger in living cells synthesized mainly from L-arginine by nitric oxide synthases (NOSs). There are three isoforms of the enzyme including endothelial (eNOS), neuronal (nNOS), and inducible NOS (iNOS) [24]. During sepsis, the infectious environment exhibits pathological characteristics different from the normal scenario, in which the elevation of NOSs expression is a distinct one. In the early phase of sepsis, the iNOS and eNOS both increased nearly 2–3 times over the physiological level [25]. On this basis, it is expected that LA, a natural NO donor with excellent biocompatibility, can liberate NO in a NOS-rich microenvironment. Therefore, the NO-donating nanoparticles designed in this context are expected to have an essential function in cardiovascular protection responding to sepsis (Fig. 1).

In summary, we have successfully designed and utilized a myocardium-targeting nanoscale carrier system that could generate NO in the heart without causing nitrate/nitrite accumulation in the circulation. In the early phase of sepsis, this targeted gas therapy efficiently improved the myocardial function by alleviating the inflammatory response, reducing ROS production, as well as protecting myocardial mitochondria. This strategy is very promising to be applied as a timely treatment to ameliorate the septic microenvironment and enhance antibiotic efficacy, thus boosting the cardioprotective effects during sepsis.

## Results

### Characterization of PCM-MSN@LA

**Fig. 2:**
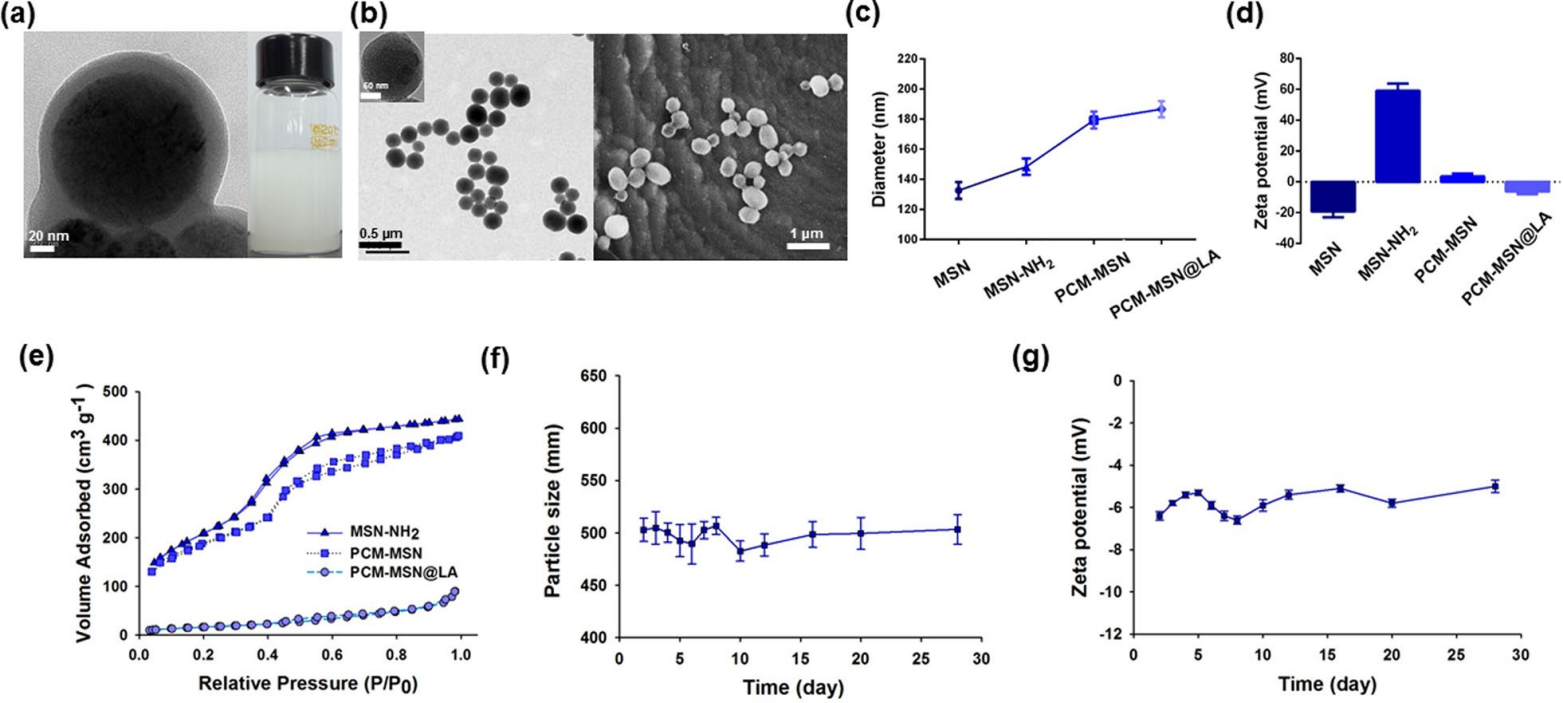
Physicochemical characterization of PCM-MSN@LA. **a** TEM images of PCM-MSN@LA particles and the morphology of PCM-MSN@LA particles (scale bar = 20 nm). **b** TEM and SEM images of PCM-MSN@LA particles (TEM: scale bar = 0.5 μm; the left upper corner image: scale bar = 50 nm; SEM: scale bar = 1 μm). Variation of MSNs after each modification in **c** size and **d** zeta. **e** The specific surface area of different samples analyzed by N_2_ adsorption–desorption method. **f**, **g** Variation of particles in size and zeta of PCM-MSN@LA in deionized water from 0 to 28 days measured using DLS

PCM-MSN@LA is featured as spherical nanoparticles (Fig. 2a) with good scattering (Fig. 2b). Conjugation of MSNs to PCM leads to increased diameters from 132.67 nm to 179.33 nm with decreased zeta potentials from -19.17 to 3.52 mV. LA loading further enlarged the particle size to 186.67 nm whereas the surface charge returned to a negative position of -6.23 mV (Fig. 2c, d). The nanoparticles showed a hexagonal array that could be indexed as (100), (110), and (200) Bragg peaks in the powder X-ray diffraction (XRD) pattern (Additional file 1: Fig. S1). Nitrogen adsorption–desorption measurements of the specific surface area of MSN-NH2, PCM-MSN and PCM-MSN@LA showed substantial difference in absorption isotherms as 764.332 m^2^ g^−1^, 661.83 m^2^ g^−1^ and 62.07 m^2^ g^−1^, respectively, indicating that some channels of mesoporous nanoparticles were occupied by PCM and filled with LA (Fig. 2e). This result demonstrated successful PCM and LA grafting to MSN nanoparticles, in which PCM and LA accounted for nearly 39.7% and 20.2%, respectively, of the total nanoparticle weight measured by TG analysis (Additional file 1: Fig. S2). In vitro stability of PCM-MSN@LA is a critic physicochemical property, which was retained at high levels without notable changes in size distribution or zeta potential during 28 days of storage at 37 °C (Fig. 2f, g).

### LA encapsulation and release profiles of PCM-MSN@LA and the cytotoxicity assay

**Fig. 3:**
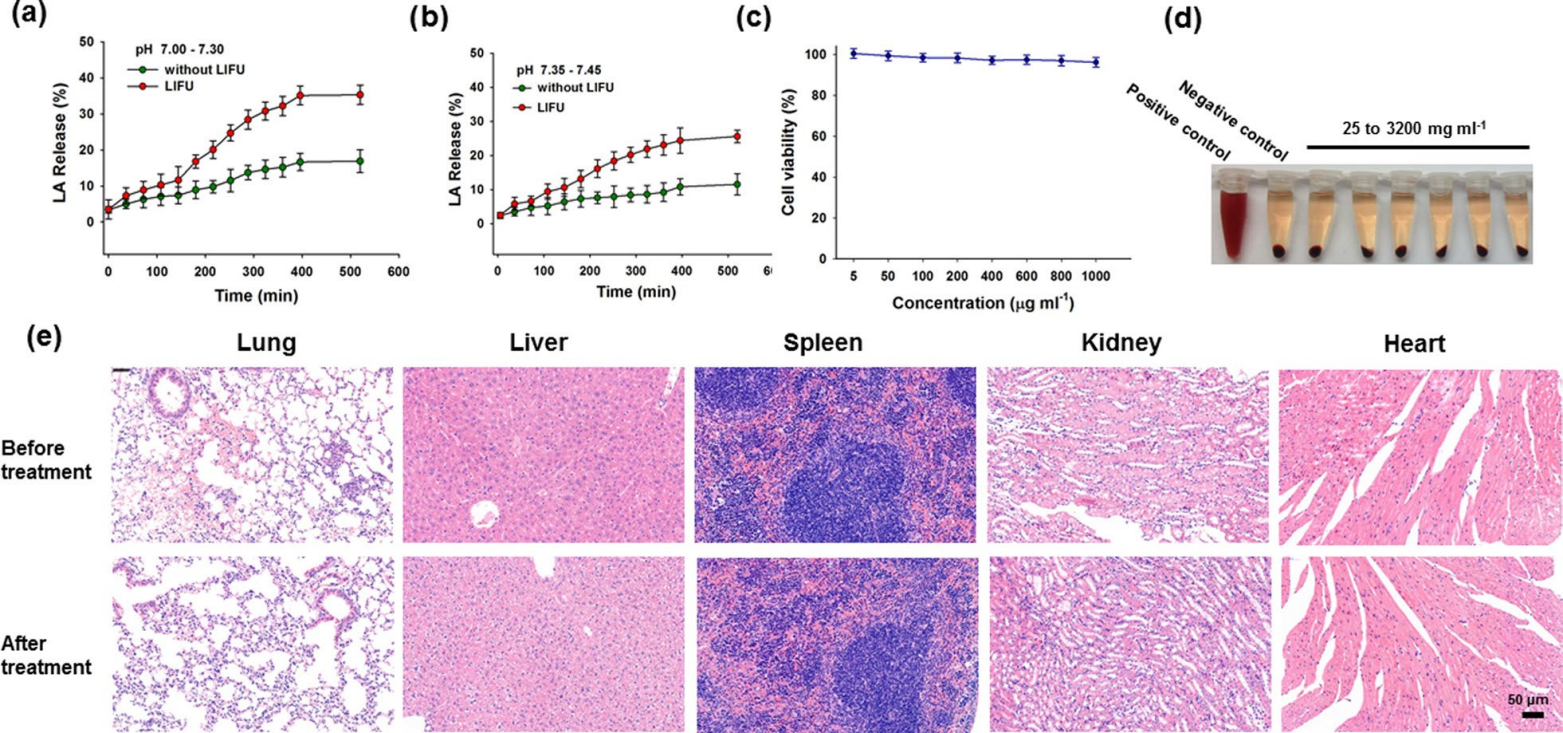
The releasing efficiency of L-arginine and safety of PCM-MSN@LA. L-arginine released from PCM-MSN@LA with or without LIFU irradiation (1.0 W/cm^2^) in HEPES with **a** PH 7.00–7.30 and **b** PH 7.35–7.45. **c** Viability of cardiomyocytes 24 h after incubation with different concentrations of PCM-MSN@LA. **d** Photographs of RBCs incubated with PCM-MSN@LA particles at different concentrations ranging from 25 to 3200 mg mL^−1^ for 3 h. The first tube and second tube were representatives of positive and negative hemolysis control respectively. **e** HE stained images of the lung, liver, spleen, kidney, and heart before and 24 h after administration of 50 mg kg^−1^ PCM-MSN@LA (scale bar = 50 μm)

Within the 10 mg of PCM-MSN@LA, there was 2.4 mg of LA in total measured by UV–vis (Additional file 1: Fig. S3), of which 11.5% and 16.9% were released in HEPES at pH 7.35–7.45 and pH 7.00–7.30, respectively, within 600 min. The release efficiency of LA was remarkably improved via LIFU to nearly 25.6% at pH 7.35–7.45 and 35.3% at pH 7.00–7.30. (Fig. 3a, b). There was no detectable cytotoxicity of PCM-MSN@LA to cardiomyocytes at concentrations below 1 mg mL^−1^ (Fig. 3c). Comparable tissue architecture in major organs, including lungs, livers, spleens, kidneys and hearts of the mice, was also observed before and 24 h after intravenous injection of 50 mg kg^−1^ PCM-MSN@LA, the 50 mg kg^−1^ PCM-MSN@LA treated mice showed minor change (Fig. 3e). Furthermore, there was no obvious hemolysis visualized after incubation of the nanoparticles with blood for 3 h at various concentrations up to 3200 mg mL^−1^ (Fig. 3d). These results demonstrate the safety profile of PCM-MSN@LA to cardiomyocytes.

### Cellular uptake of PCM-MSNs in vitro and PCM-MSN@LA + LIFU biodistribution in vivo

**Fig. 4:**
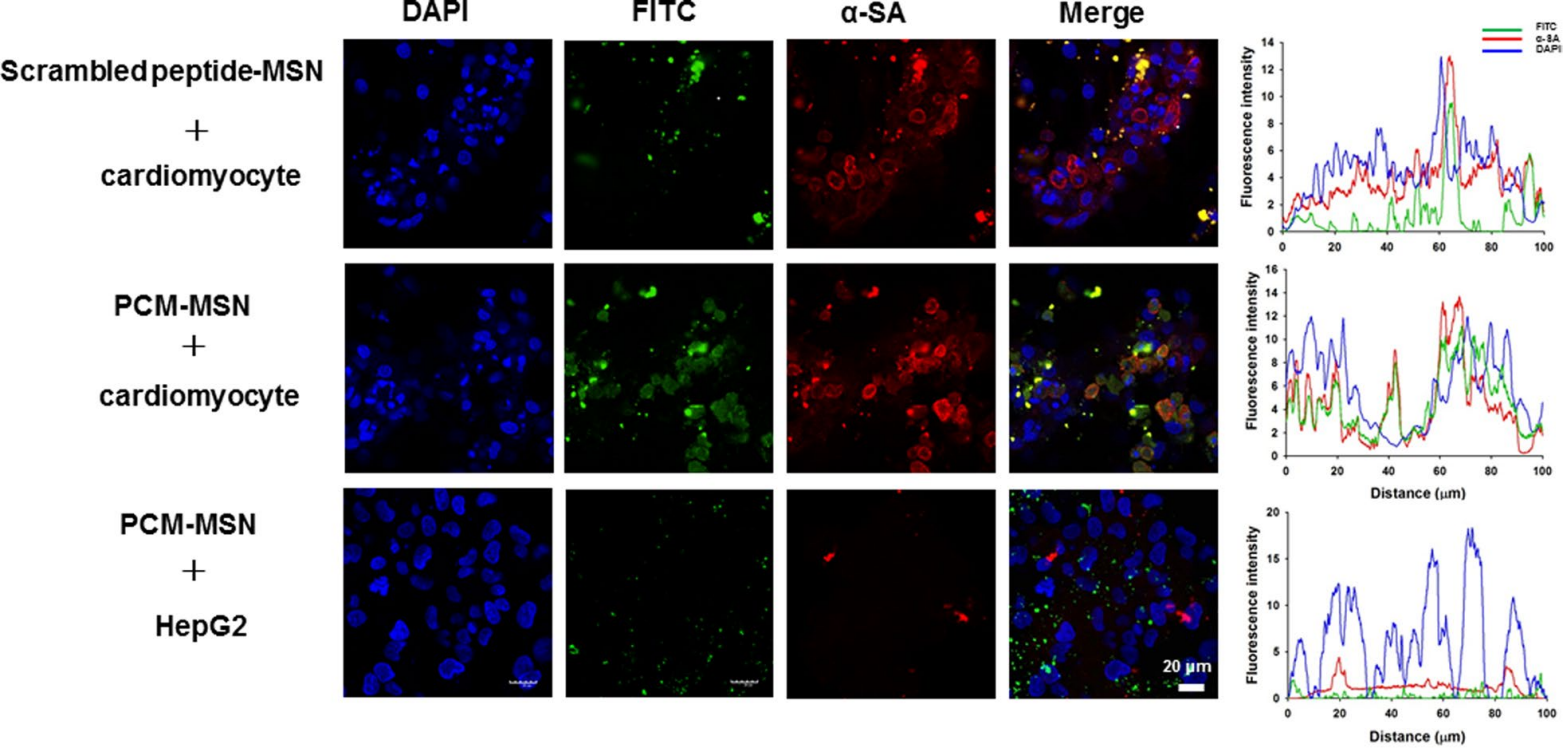
Specific affinity of PCM in cardiomyocytes in vitro. Immunofluorescence staining and fluorescence intensity of cardiomyocytes after incubated with scrambled peptide-MSN and PCM-MSN for 1 h. Immunofluorescence staining and fluorescence intensity of HepG2 after incubation with PCM-MSN for 1 h. DAPI (blue; nuclei), FITC (green; PCM or scrambled peptide), α-SA (red; cardiomyocyte) (scale bar = 20 μm)

PCM showed a high affinity for cardiomyocytes as confirmed by robust green FITC-PCM signals co-localized with red PE-α-SA signals as a cardiomyocyte-specific marker in the PCM-MSN group (Fig. 4). In contrast, there were significantly reduced green FITC signals in the scrambled peptide group with cardiomyocytes or scarce in the HepG2 groups with PCM-MSNs, suggesting the specific affinity of PCM for cardiomyocytes.

**Fig. 5:**
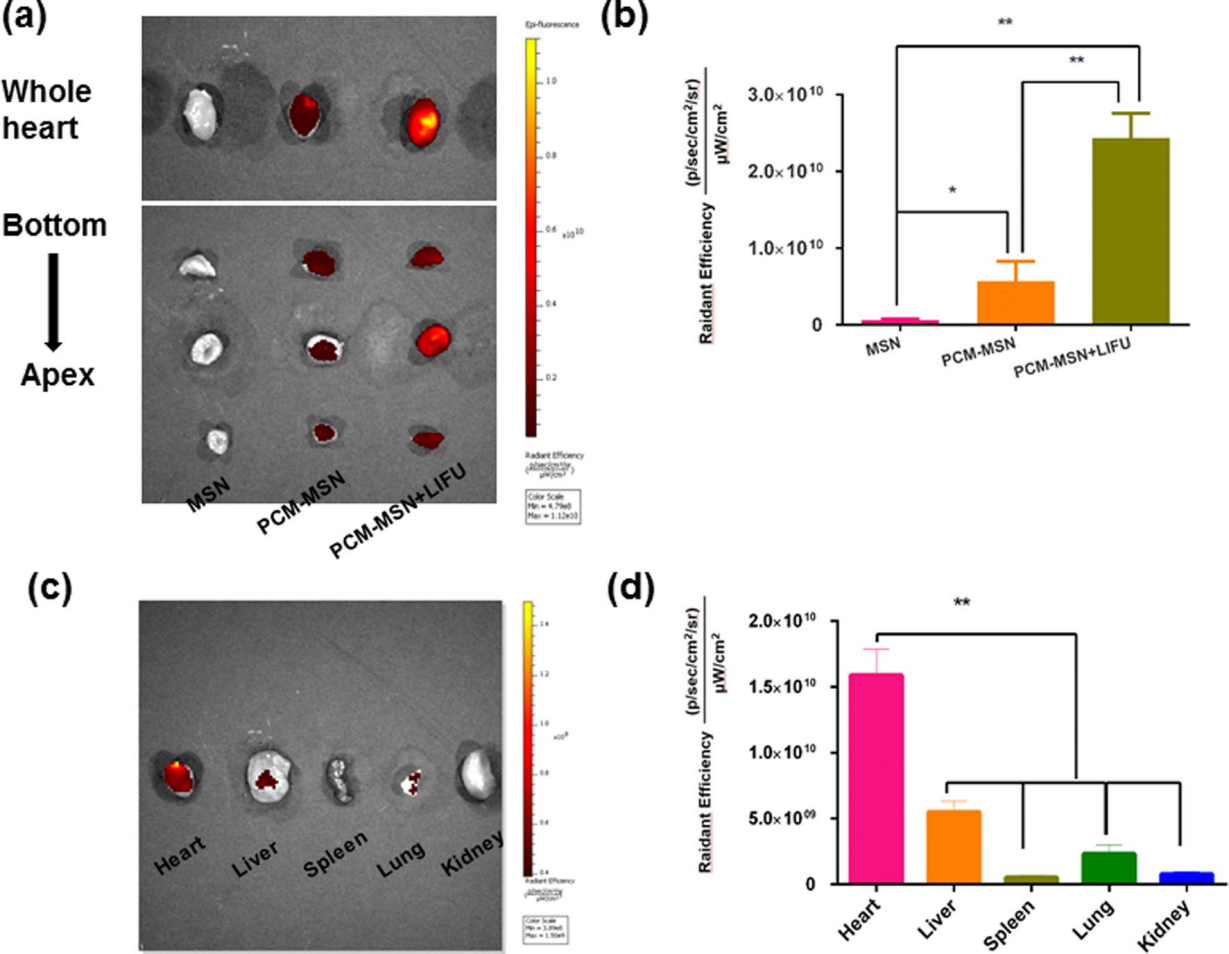
Specific cardiac targeting and delivery of PCM-MSN + LIFU in vivo. **a** Immunofluorescence study showing delivery efficiency of Cy5.5-stained MSN or PCM-MSN with or without LIFU to the heart at 6 h after individualized treatments. **b** Fluorescence intensity analysis after individualized treatments (n = 4). Data were presented as mean ± SD. *P < 0.05; **P < 0.01. **c** Accumulation of Cy5.5-stained PCM-MSN observed in the heart and other organs after LIFU irradiation. **(d).** Quantitation of merged fluorescence intensity in different organs (n = 4). Data were presented as mean ± SD. **P < 0.01

The transpotation capability and efficiency of PCM to the heart with or without LIFU were assessed in vivo. Red fluorescence signals representing nanoparticles were visualized in multiple major organs. In comparison to the control MSN group, there were significantly increased signals, with nearly 60-fold higher fluorescence intensity, in the heart of mice with PCM-MSN. It indicates PCM mediated efficient transportation of MSN targeting the heart. The targeting efficiency was further boosted via LIFU with a sevenfold increase of fluorescence intensity visualized in the PCM-MSN + LIFU group compared to the PCM-MSN one (Fig. 5a, b). LIFU irradiation enables PCM-MSN to penetrate through cardiac microvessels more easily to reach the myocardium. This effect is specific to the heart as most other organs or tissues, including the liver, kidneys, lungs and spleen, showed substantially less distribution of PCM-MSN (Fig. 5c, d). Altogether it demonstrates that the combination of PCM and LIFU strategies ensured efficient nanoparticle transportation to the heart and efficiently improved the delivery to cardiomyocytes.

### NO production from the myocardium-targeted NO release system in cardiomyocytes

**Fig. 6:**
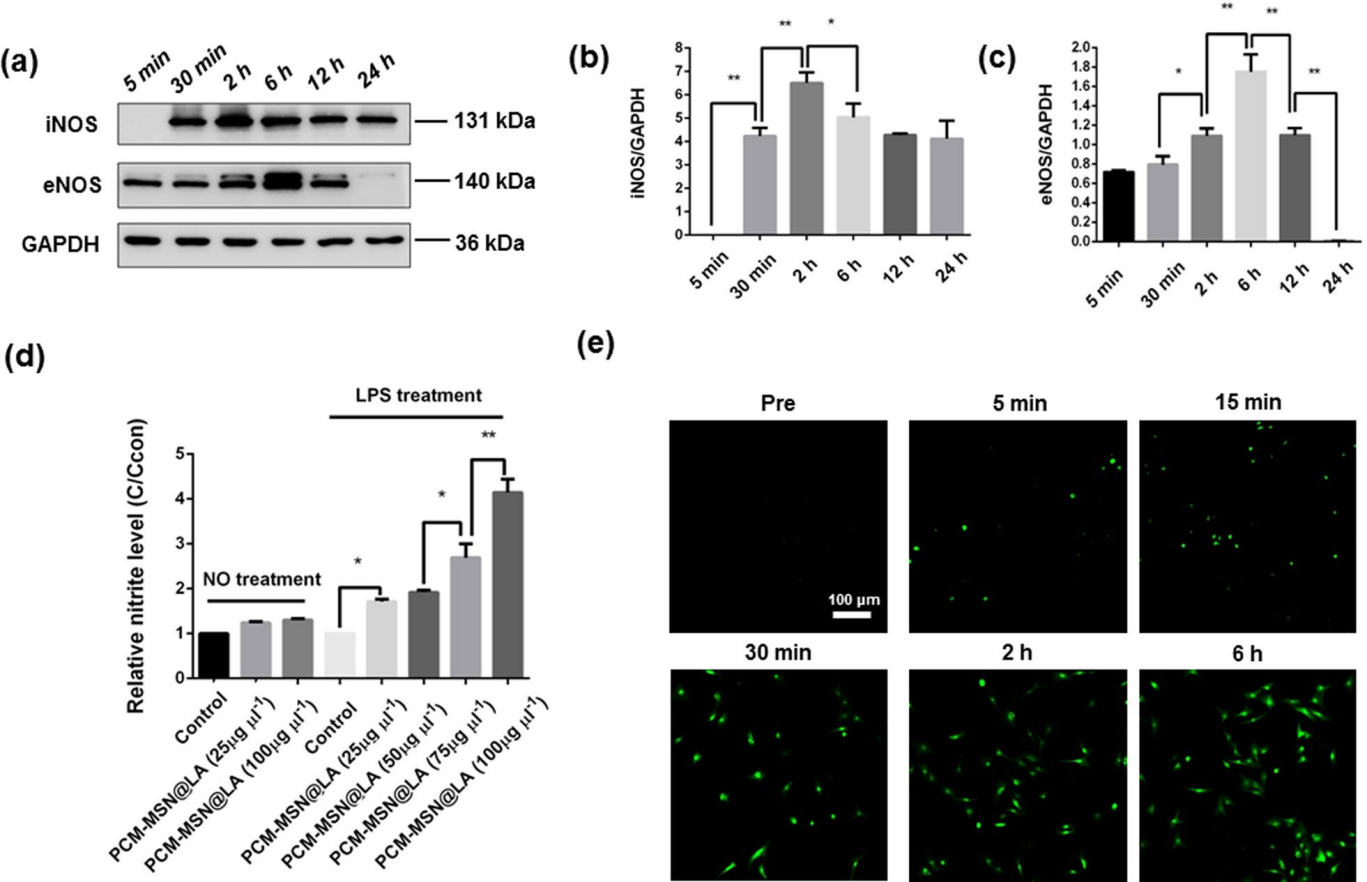
Assessment of NO generation from PCM-MSN@LA in the cardiomyocytes with or without LIFU in vitro. **a** The expression of iNOS and eNOS in LPS-incubated cardiomyocytes from 5 min to 24 h. **b**, **c** The quantitative analyses of iNOS and eNOS expression (n = 6). Data were presented as mean ± SD. *P < 0.05; **P < 0.01. **d** Relative nitrite concentration from different doses of PCM-MSN@LA in cardiomyocytes with or without LPS (n = 6), Data were presented as mean ± SD. *P < 0.05; **P < 0.01. **e** Confocal images of cardiomyocytes stained by DAF-FM DA following 75 μg mL^−1^ PCM-MSN@LA irradiated with LIFU over time (scale bar = 100 μm)

NO synthase is required to generate NO in vivo. Upon LPS stimulation, the endogenous expression of NOS isoforms, iNOS and eNOS, was remarkably elevated in cardiomyocytes from 30 min up to 12 h with peaks at 2 h and 6 h, respectively (Fig. 6a–c).

The NO production capacity of PCM-MSN@LA in combination with LIFU in cardiomyocytes was further studied by measuring NO levels through Griess assay and fluorescence microscopy after DAF-FM DA staining. There was no obvious NO increase in cardiomyocytes with PCM-MSN@LA in the absence of LPS treatment, which levels were similar to the untreated cells. On the contrary, the nitrite levels were significantly upregulated in the presence of LPS treatment in a dose-dependent manner (Fig. 6d). Likewise, DAF-FM DA staining detected substantially enhanced green fluorescence signals, which represented NO production in LPS-induced cardiomyocytes, in the presence of 75 μg mL^−1^ PCM-MSN@LA combined with LIFU within 6 h, further supporting the evidence of NO release (Fig. 6e). These data indirectly suggest that PCM-MSN@LA could produce NO effectively in the context of infection. Interestingly, although extraordinary upregulation was observed after LPS treatment, the absolute NO levels remained low in the cardiomyocytes with PCM-MSN@LA. The absolute nitrite level was 0.5 μM 2 h after LPS stimulation, even co-incubated with PCM-MSN@LA, the absolute nitrite level was 0.56 μM only. This low concentration is critical for the influence of NO on the heart to maintain cardiovascular homeostasis [24].

### PCM-MSN@LA alleviates LPS-induced injury in cardiomyocytes

**Fig. 7:**
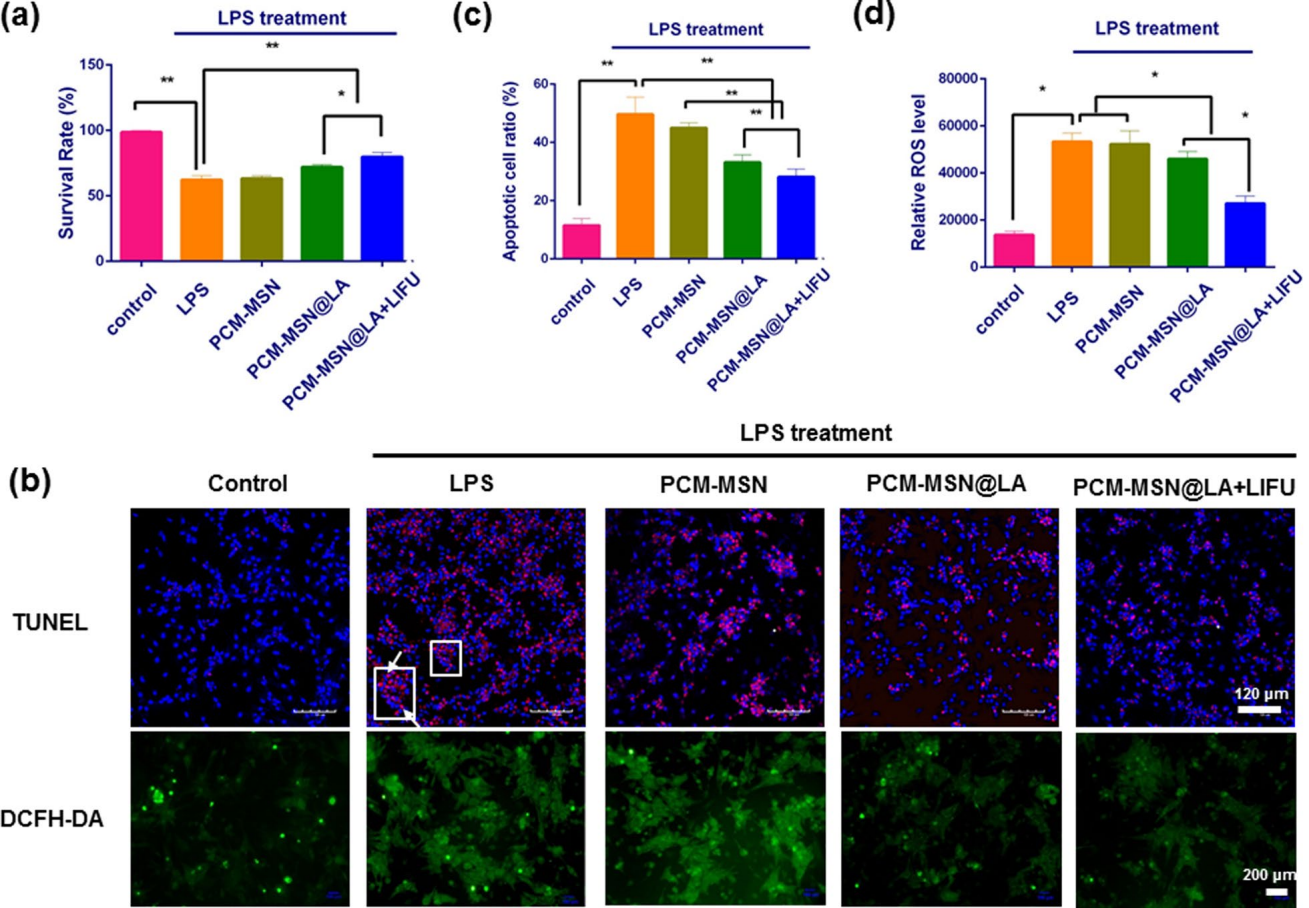
PCM-MSN@LA reduced cardiomyocyte apoptosis. **a** The viability of cardiomyocytes isolated from each group with different treatments (n = 6). Data were presented as mean ± SD. *P < 0.05; **P < 0.01. **b** Photomicrographs of cardiomyocyte apoptosis by TUNEL staining (scale bar = 120 μm) and ROS burden by DCFH-DA staining (scale bar = 200 μm) after individualized treatments. DAPI (blue; nucleic); TUNEL positive (red; apoptotic nucleic; white arrow); ROS positive (green). **c** Quantitative analysis of TUNEL positive cells which were representative of apoptosis (n = 6). Data were presented as mean ± SD. *P < 0.05; **P < 0.01. **d** Quantitative analysis of intensity of ROS fluorescence treatments (n = 6). Data were presented as mean ± SD. *P < 0.05; **P < 0.01

LPS is able to induce massive damage in tissues and organs. This effect can be alleviated by PCM-MSN@LA, and, to a further extent, by PCM-MSN@LA + LIFU in cardiomyocytes with the cell viabilities improved from 62 to 72% and 80%, respectively (Fig. 7a). Evaluation of apoptosis by the CCK-8 assay and TUNEL staining revealed severely injured cardiomyocytes induced by LPS with or without PCM-MSN (Fig. 7b). Nevertheless, the percentage of apoptotic cells has been substantially decreased by PCM-MSN@LA, and to a greater extent, by PCM-MSN@LA + LIFU (Fig. 7c). To confirm the effect of LIFU-mediated PCM-MSN@LA on ROS scavenging, the intracellular ROS probe, DCFH-DA, was used. As shown in Fig. 7d, the average ROS level in cardiomyocytes treated with LPS has decreased after PCM-MSN@LA incubation with or without LIFU for 6 h. However, this reduction was not observed in cells with PCM-MSN only. This finding suggested that ROS reduction was potentially associated with NO release in the cardiomyocytes.

### Myocardium-targeted NO release alleviated cardiac apoptosis and improved survival rate during sepsis

**Fig. 8:**
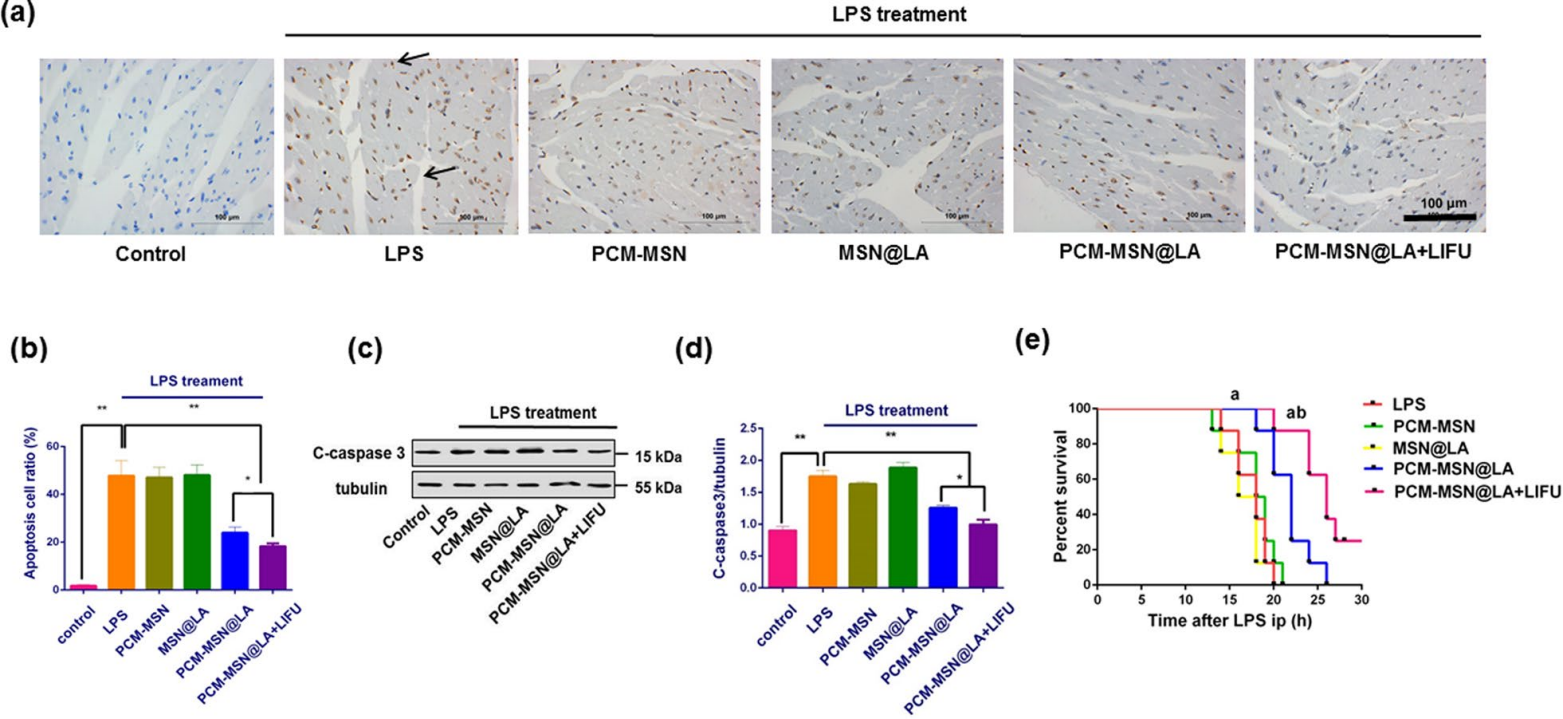
Myocardium-targeted NO release significantly attenuated LPS-induced apoptosis and decreased the expression of caspase 3 in cardiac tissue. **a** Representative TUNEL staining of cardiac sections from mice with different treatments. DAPI (blue; nuclei); TUNEL positive (brown; black arrow) (scale bar = 100 μm). **b** Quantitative analysis of apoptotic cells (n = 8). Data were presented as mean ± SD, *P < 0.05; **P < 0.01. **c**, **d** The expression and quantitative analysis of caspase 3 protein (n = 8). Data were presented as mean ± SD. *P < 0.05; **P < 0.01. **e** Survival analysis of mice with severe sepsis and different treatments (n = 6). ^a^*P* < 0.05 vs. CLP; ^b^*P* < 0.05 vs. PCM-MSN@LA

The protective effect of targeted NO release on injured myocardium during LPS treatment was evaluated by the TUNEL assay. Numerous necrotic cells in brown were detected across the cardiac tissue upon LPS administration (Fig. 8a). The degree of necrosis was effectively lessened by PCM-MSN@LA, and to a greater degree by PCM-MSN@LA + LIFU, yet not by PCM-MSN or MSN@LA (Fig. 8a, b). Simultaneously, the levels of caspase-3 protein, which is an apoptotic index with upregulated expression during necrosis, displayed a similar tendency (Fig. 8c, d). The cardiac function is closely related to the survival rate. Survival analysis confirmed the highest survival rate in the PCM-MSN@LA + LIFU group, followed by the PCM-MSN@LA group to a lesser extent (Fig. 8e). These data indicate that a precise and efficient release of LA at a low level could facilitate the protection of the myocardium in the sepsis model.

### Cardiovascular protection by localized NO release during sepsis

To verify whether precisely targeted NO release could prevent heart dysfunction during sepsis, several serum markers including cTnI, CK-MB, and LDH were measured. In addition, the echocardiography (ECG), blood pressure, and histological morphology of all animals were examined.

**Fig. 9:**
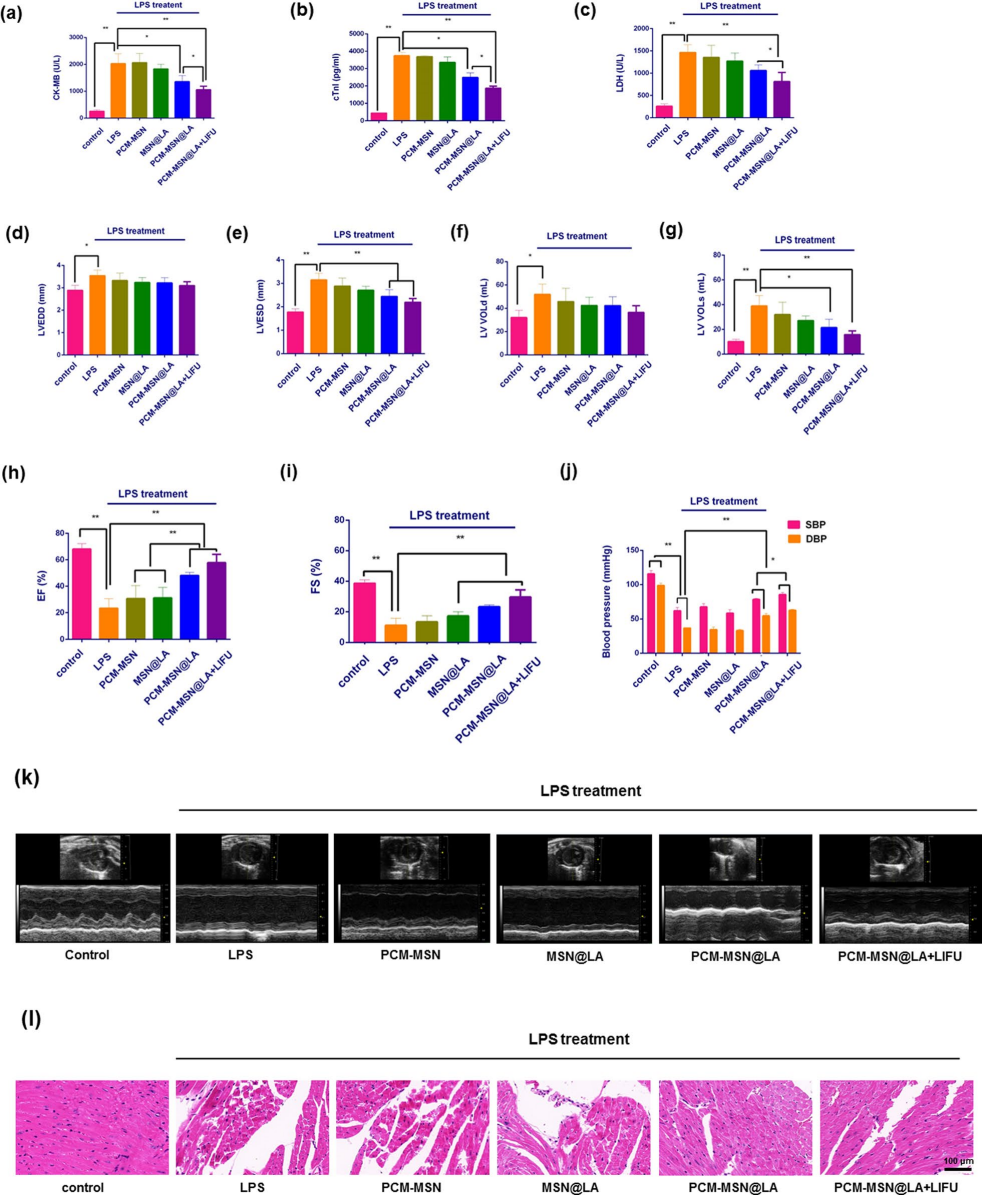
Targeted NO release in the myocardium prevented heart dysfunction. **a**–**c** The expression level of clinical markers in the serum of different groups (n = 8). **d**–**i** Echocardiographic assessment of LV functions (n = 8). Data were presented as mean ± SD, *P < 0.05; **P < 0.01. LVEDD, left ventricular internal diameter at the end of diastole; LVESD, left ventricular internal diameter at the end of systole; LV VOL d, left ventricular volumes at the end diastole; LV VOL s, left ventricular volumes at the end systole; EF, ejection fraction; FS, fraction shorting. **j** Blood pressure measurements of the animals (n = 8). Data were presented as mean ± SD, *P < 0.05; **P < 0.01. SBP, systolic blood pressure; DBP, diastolic blood pressure. **k** Representative M-mode echocardiograms presented in the parasternal short-axis view (at the level of papillary muscle) for FS and EF measurements following different treatments. **l** Representative morphological analysis by H&E staining (scale bar = 100 μm)

There was a ninefold higher expression of cTnI and CK-MB (3739.9 ± 182.3 vs. 430.4 ± 27.2; 2026.3 ± 370.2 vs. 253.9 ± 57.6), and a sixfold increase in LDH expression (1344.4 ± 240.5 vs. 259.9 ± 50.5), in the serum of the mice injected with LPS, as compared to the control group of normal mice. Treatments with PCM-MSN or MSN@LA had no apparent effects on the expression of these markers compared with the LPS group. Nevertheless, PCM-MSN@LA injection, especially combined with LIFU irradiation, notably dropped the levels of these markers with the reduction rates of 37%, 31% and 21% in the former group, and 53%, 47% and 39% in the other one (Fig. 9a–c) for cTnI, CK-MB, and LDH, respectively.

Along with the changes in the serum markers, ECG analysis showed significantly higher EF and FS, whereas lower LV VOLs and LVESD, in the PCM-MSN@LA-treated mice with slightly better parameters in the mice receiving LIFU simultaneously, whereas no much difference in the other groups (Fig. 9d–i). This was also observed via the M-mode in the short-axis view (Fig. 9k).

Edema and inflammatory cell infiltration accompanied with disorganized myocardial fibers were presented in the hearts of the LPS group, as shown by H&E analysis (Fig. 9l). The degree of edema and fibrosis in the hearts was substantially relieved in the presence of PCM-MSN@LA and PCM-MSN@LA + LIFU. This suggested that the precise location of LA to the heart followed by its conversion to NO resulted in cardiac protection in the sepsis mouse model.

Hypotension is another major hemodynamic disorder that is closely linked to sepsis-related mortality. All septic mice exhibited lower blood pressure than the controls despite of fluid resuscitation. The PCM-MSN treatment did not recover the blood pressure or, even worse, the MSN@LA aggravated this disorder in related animals. PCM-MSN@LA, particularly plus LIFU, significantly mitigated LPS-induced hypotension (Fig. 9j). Accordingly, this cardiovascular recovery was not associated with other vasodilatory side effects in the animals. Thus, this elaborately designed strategy of PCM-MSN@LA in combination with LIFU irradiation could efficiently alleviate myocardial dysfunction in the sepsis model.

### The mechanism of myocardium-targeted NO release on LPS-induced cardiac dysfunction

The potential mechanism of in vivo myocardium protection by PCM-MSN@LA + LIFU during sepsis can be attributed to the following three reasons: (1) regulating the myocardial inflammatory response; (2) ameliorating oxidant stress; (3) maintaining mitochondrial function. These mechanisms were investigated in this context.

**Fig. 10:**
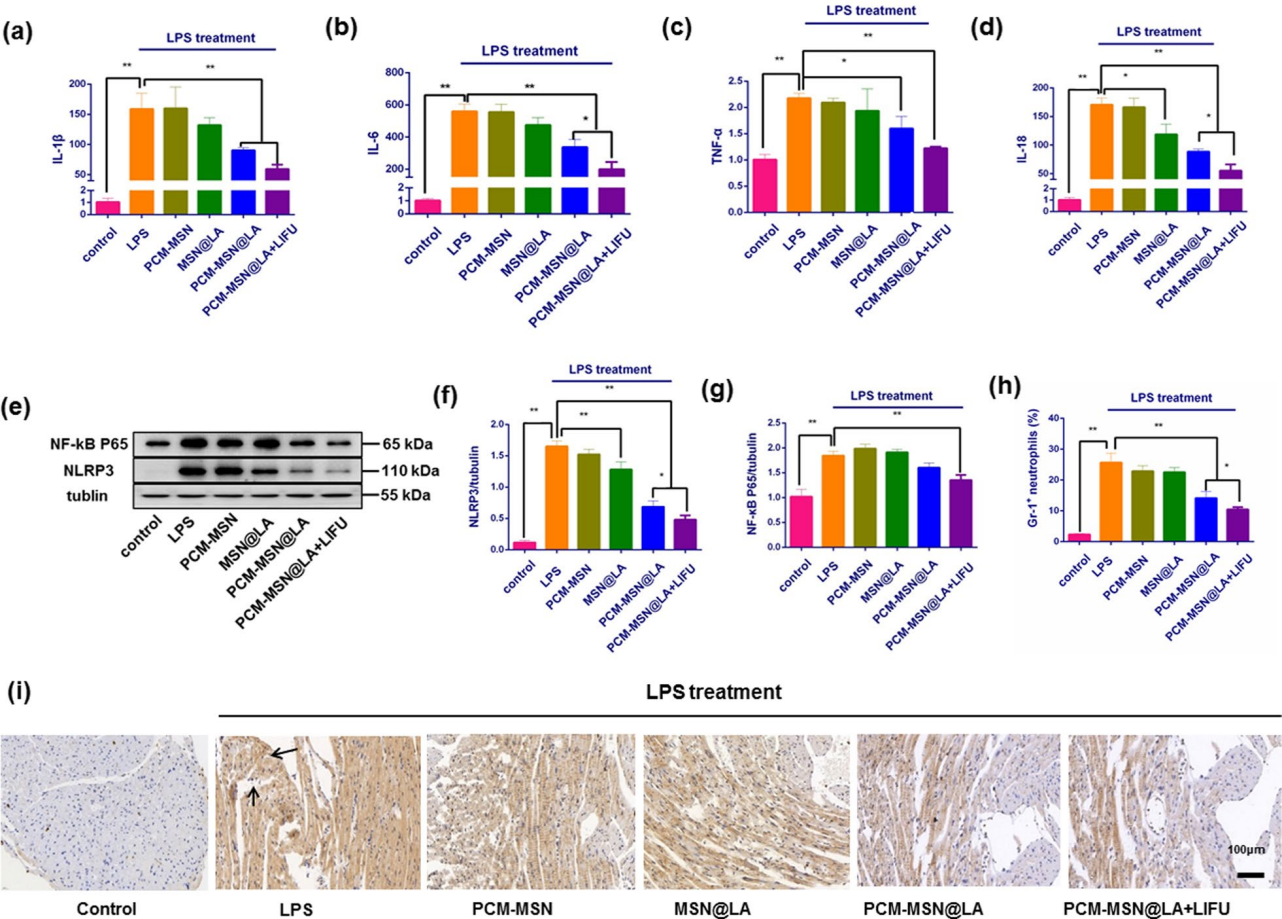
Myocardium-targeted NO release effectively relieved LPS-induced myocardial pro-inflammatory response. **a**–**d** Inflammatory cytokines including IL-1, IL-6, IL-18, and TNF-α in serum measured 6 h after different treatments (n = 8). Data were presented as Mean ± SD. *P < 0.05; **P < 0.01. **e** The expression of inflammatory mediator proteins NLRP3 and NF-κB P65. **f**, **g** Quantitative analysis of NLRP3 and NF-κB P65 expression (n = 8). Data were presented as Mean ± SD. *P < 0.05; **P < 0.01. **h** The percentage of Gr-1^+^ cells in the heart tissue (n = 8). Data were presented as Mean ± SD. *P < 0.05; **P < 0.01. **i** Immunohistochemical analysis of cardiac tissue slices with Gr-1^+^ staining. DAPI (blue; nuclei); Gr-1^+^ positive (brown; black arrow) (scale bar = 100 μm)

The general effects of LPS in the body have been described as overwhelming accumulation of inflammatory cytokines including IL-1β, IL-6, IL-18 and TNF-α, and upregulated expression of inflammatory-associated proteins including NLRP3 and NF-kB P65. The same actions have been also observed in the animals treated with LPS in this context (Fig. 10a–g). Delivery of PCM-MSN@LA, especially under LIFU irradiation, significantly eased extensive inflammatory response in the heart with statistically significant reduction of IL-1β, IL-6, IL-18 and TNF-α mRNA, as well as NLRP3 and NF-kB P65 protein expression. Similar changes were observed in Gr-1 expression, which is associated with the functional performance of neutrophils. There was large amount of Gr-1^+^ neutrophils with brown nuclei recruited to the myocardium of LPS-treated mice. The amount of Gr-1^+^ neutrophils was also largely decreased in the groups receiving PCM-MSN@LA and PCM-MSN@LA + LIFU, yet remained almost the same in those with other treatments. It indicates the PCM-MSN@LA + LIFU remarkably limited the accumulation of functional neutrophils in the myocardium in the sepsis animals (Fig. 10h, i).

**Fig.11:**
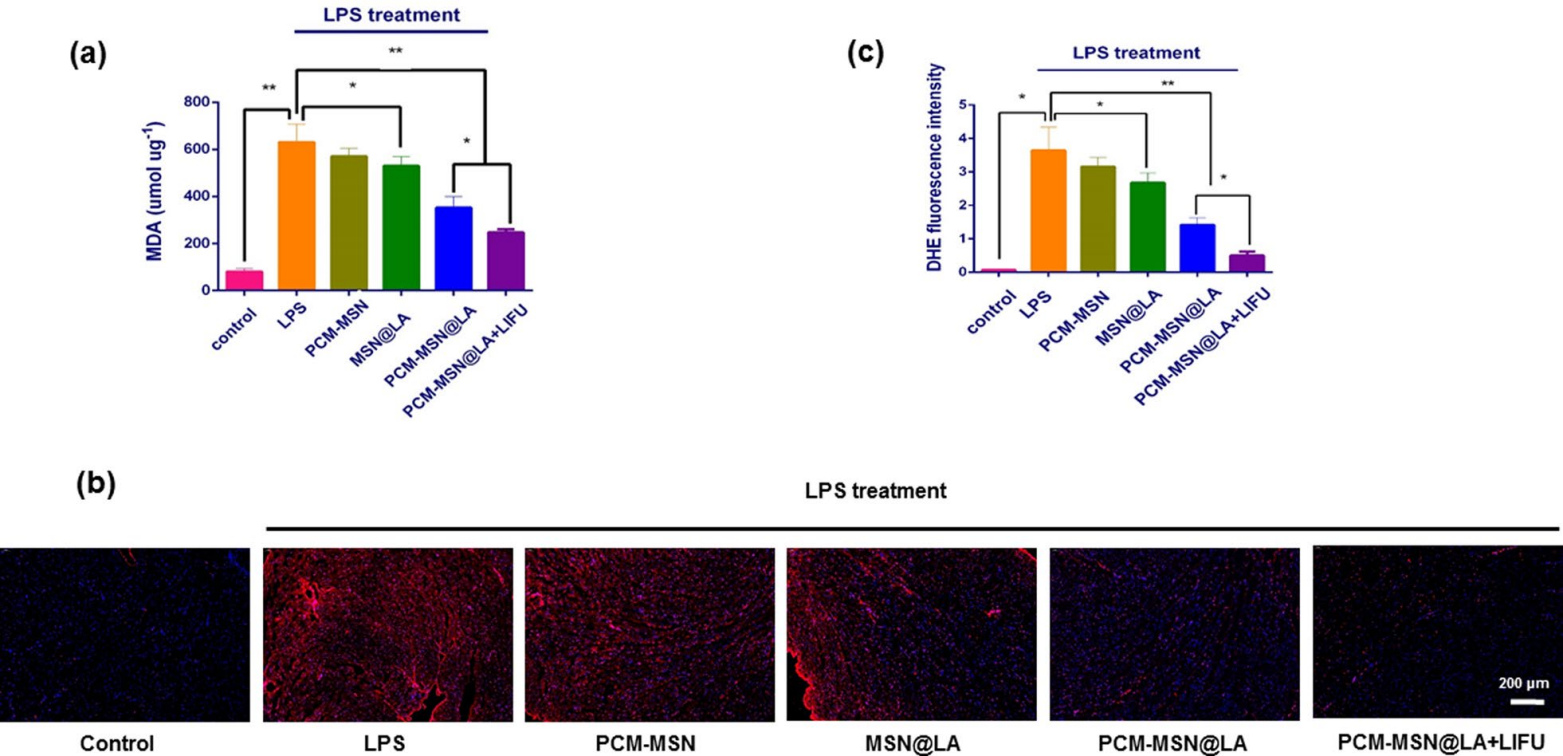
Myocardium-targeted NO release effectively relieved LPS-induced myocardial antioxidants stress. **a** Measurement of MDA concentration (n = 8). Data were presented as mean ± SD, *P < 0.05; **P < 0.01. **b** Representative image of DHE stained heart sections with a ROS fluorescent probe. DAPI (blue, nuclei); ROS positive (red) (scale bar = 200 μm). **c** Quantitative analysis of ROS fluorescent intensity (n = 8). Data were presented as mean ± SD, *P < 0.05; **P < 0.01

ROS generation was assessed using MDA and DHE analyses. LPS-induced elevation of MDA levels in the cardiac was decreased by PCM-MSN@LA with statistical significance (Fig. 11a). Oxidative stress in the myocardium measured by DHE also showed a concomitant increase in LPS treated mice, which was significantly suppressed by PCM-MSN@LA. Moreover, both ROS and oxidative stress activities were the least in all groups with different treatments. Collectively, PCM-MSN@LA + LIFU exhibited excellent antioxidant activity against the ROS burden after myocardial injury in the LPS-induced sepsis model (Fig. 11b, c).

**Fig. 12:**
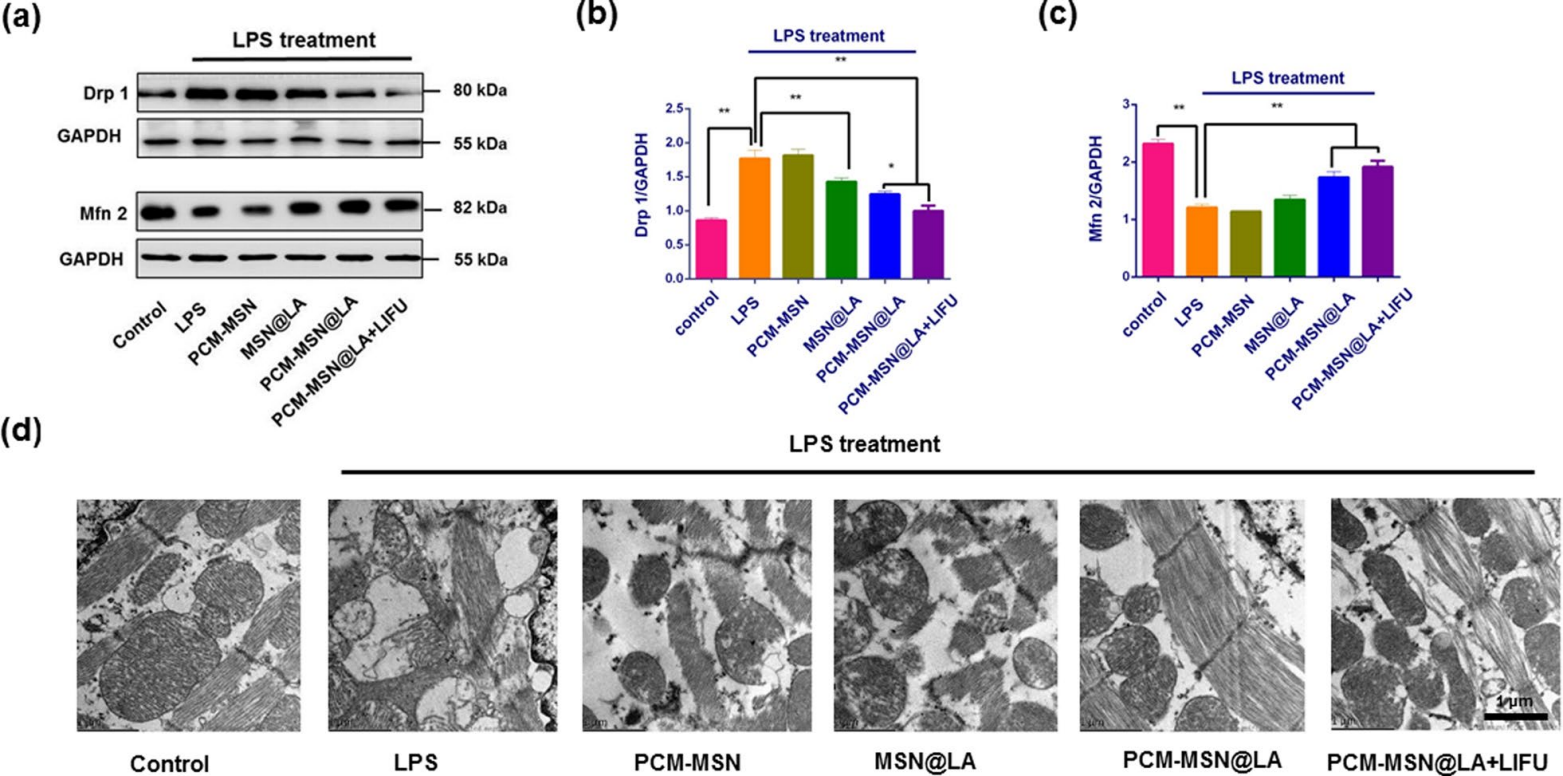
Myocardium-targeted NO release effectively prevented mitochondrial dysfunction in the myocardium of the LPS-induced sepsis model. **a** Expression of mitochondrial mediator proteins Drp1 and Mfn 2. **b**, **c** Quantitative analysis of Drp1and Mfn 2 expression (n = 8). Data were presented as mean ± SD, *P < 0.05; **P < 0.01. **d** TEM analysis of myocardial sections (scale bar = 1 μm)

Drp1 and Mfn 2 are two regulatory enzymes that control mitochondrial fission and fusion [26, 27]. They are closely related to mitochondrial function and can be affected by LPS with upregulated Drp1 and downregulated Mfn 2 levels in LPS-treated mice. As expected, these dysregulations can be reversed by PCM-MSN@LA, and LIFU to a greater extent, yet not by PCM-MSN or MSN@LA injections (Fig. 12a–c). Consistent with the dysregulated enzyme levels, impaired mitochondria structure of various sizes, vacuolar changes and partial cristae disruptions, along with swollen damaged cardiac muscle fibres, were further investigated in LPS group by TEM analysis (Fig. 12d). These structural impairments were substantially attenuated only in the mice with PCM-MSN@LA, particularly in the presence of LIFU irradiation, suggesting that PCM-MSN@LA + LIFU could effectively restore mitochondrial function, probably via efficient release of NO in the cardiac.

## Discussion

Cardiac dysfunction is an early and fatal complication of sepsis [12]. Although there has been great interest in the development of NO therapy in the treatment of cardiovascular diseases, the therapeutic NO strategy for modulation of myocardial function in sepsis has not been practically used yet, due to the rapid diffusion of NO as a major drawback [28]. We have developed a non-toxic and efficient NO donor delivery system, PCM-MSN@LA + LIFU, to precisely control NO release during sepsis so as to protect cardiac function from LPS injury. This is the first time to report the efficiency and safety of a targeted NO donor delivery system in the septic model.

The targeted delivery of NO is based on a MSN-conjugated 20-mer peptide, namely PCM, with an excellent binding capacity to the heart. I*n vitro* study in cell lines and in vivo study using different tissues both demonstrated the specific affinity of PCM-MSN to cardiomyocytes as compared to the scramble control. The strong PCM-MSN signal distributed in the mouse heart, which was threefold weaker in the control animals, suggested that PCM had efficiently guided nanoparticles to the heart. Thus it enhanced tissue-specific uptake of MSN as consistent with previous publications [15, 16, 29, 30].

McGuire et al. [15] developed a quantitative real-time PCR assay to show that the PCM had a nearly 50-fold increase in cardiac selectivity over the control 20-mer peptides. Apart from the high selectivity, other advantages of peptides include lower molecular weight, easier synthesis, lower cytotoxicity and better conjugation capability onto nanocarriers compared to antibodies [14]. On the other hand, a LIFU-triggered drug delivery system could be a potential solution for liberating a drug from its formulation at a focused site. Ultrasound with high US energy, which is also called high-intensity focused US (HIFU), only induced limited delivery to the heart. Conversely, noninvasive LIFU with low energy exhibited considerable tissue-penetrating ability. Thus, it has been intensively studied for therapy and diagnostic imaging purposes [16–18, 31]. Consistent with an optimised frequency, we have set 1.0 W/cm^2^-20% power as the LIFU parameter [19]. Indeed, LIFU irradiation facilitated successful targeting and delivery of PCM-MSN@LA to the cardiac tissue resulting in more NO release yet without inducing prominent side effects on the circulation.

There is another concern with the level of NO production. NO is produced in various types of cells by NOS isoforms through the oxidation of LA. NO deficiency is critically associated with the onset and development of cardiovascular disease [10, 32]. Our results of NO generation in situ demonstrated that different doses of PCM-MSN@LA, particularly under the LIFU irradiation, induced relative changes in NO levels in cardiomyocytes compared to other treatments. Under normal circumstances, there is only a low expression of eNOS without any significant modulation in NO generation. In the situation of sepsis, NO generation related to PCM-MSN@LA was closely associated with increased levels of iNOS and eNOS, indicating that the nanoparticles mediated NO generation in this specific context, which in turn acted on the body. Secondly, the data from the UV–vis assay showed that with exposure to LIFU irradiation, 25.6% of LA release from PCM-MSN@LA nanoparticles in pH 7.35–7.45 solutions 35.3% in pH 7.00–7.30 solutions. This confirms the absolute NO level generated from the PCM-MSN@LA + LIFU treatment in the acidic microenvironment of sepsis was not excessive. The biological effects of NO highly relied on the absolute concentration of NO [33]. The absolutely low level of NO was associated with some of the most important immunopathologies in cardiovascular diseases with a direct influence on cardiovascular dysfunction [10, 25, 34]. Thus, the sequential generation system of NO responsive to the septic microenvironment showed a very promising therapeutic strategy for the heart in this context.

In addition, our data clearly showed the central role of NO generated from the nanoparticles in inhibiting cardiomyocyte apoptosis and ameliorating cardiac function in severe sepsis. The therapeutic effects of NO-releasing nanoparticles in the sepsis scenario could be attributed to several mechanisms including immune regulation, ROS reduction and mitochondrial modulation. Excessive cytokines and inflammatory factors induced by LPS had been remarkably decreased by the PCM-MSN@LA + LIFU treatment, which was consistent with previous research [35, 36]. NLRP3 and NF-κB P65 are two important components of the inflammasome. Stimulation of NF-κB P65 together with NLRP3 activation led to a lethal consequence of cardiac dysfunction [37]. Thus, downregulation of NLRP3 and NF-κB P65 expressions with simultaneous inhibition of proinflammatory cytokines could effectively improve heart function in the sepsis model. The observation of much fewer Gr-1^+^ neutrophils in the myocardium further confirmed that NO played a central role in neutrophil migration in the presence of PCM-MSN@LA + LIFU.

LPS leads to the worsening of oxidative stress, which promoted proinflammatory immune reactions and severe damage to mitochondrial metabolism [7, 38]. The reduction of NO bioavailability was associated with excessive ROS generation which was also witnessed in this context. We assumed that NO production was capable to recover the balance with retained bioactivity. Consequently, bioactive NO could modulate NADPH oxidase and regulate cardiac mitochondrial respiration to protect the heart away from neutrophil infiltration and further mitigate the ROS burden [25, 39–42]. As predicted, the application of PCM-MSN@LA + LIFU liberated bioactive NO in the myocardium, exhibiting a robust inhibitory effect on LPS-induced oxidative stress as was reflected by reduced MDAs levels and significantly decreased DHE-positive cells in the sepsis animals.

The high mitochondrial density allows instant and fast energy production in cardiomyocytes [4]. Mitochondrial dysfunction in sepsis was manifested as elevated Drp1 expression and diminished Mfn2 expression. PCM-MSN@LA + LIFU therapy showed extensive mitochondrial protection by diminishing the Drp1 as well as elevating the Mfn2 expression. In addition, the morphology and density were also much improved after PCM-MSN@LA + LIFU treatment as confirmed by TEM analysis.

Despite decades of research regarding the effects of NO donor in sepsis, there has been only limited clinical application of NO treatment for cardiac sepsis. One caveat is that the vasodilatory action induced by NO could further accelerate hemodynamic collapse [20]. Notably, unlike MSN@LA which predominantly lowered the blood pressure, PCM-MSN@LA, in response to LIFU irradiation, quickly entered and accumulated in the septic cardiomyocytes, whereas, at the same time avoided the collapse of blood pressure. Thus, precise myocardium-targeting of NO-producing nanoparticles could successfully avoid the side effects of NO diffusion during myocardial protection.

Unfortunately, it is still challenging to monitor the ultrasound imaging-guided gas delivery in vivo with currently available technologies. The comprehensive process of NO liberation to the heart remains to be investigated and addressed in future.

## Conclusions

Overall, the myocardial delivery of NO donor was accomplished by the PCM conjugation plus LIFU guided strategy designed and described in this context. Responding to the infective microenvironment, the PCM-MSN@LA executed a successful generation of NO at a balanced low level in the heart. This precise gas therapy showed excellent benefits in myocardial recovery from sepsis. Our design provides a novel therapeutic strategy with great promise to treat sepsis-induced myocardial injury.

## Methods

### Synthesis of PCM-MSN@LA

3-aminopropyltrietho xysilane (APTS), N-Cetyltrimethylammonium bromide (CTAB, 99%), N-hydroxysuccinimide (NHS, 98%) and 1-ethyl-3-(3-dimethylaminopropyl) carbodiimide (EDC, > 97%) were purchased from Alfa Aesar (Tianjin, China). HCl solution (37%) and sodium hydroxide (NaOH) were obtained from Xilong (Guangdong, China). The FITC-labeled PCM and scrambled peptide ligands were synthesized by Sangon Biotech Co., Ltd (Shanghai, China) as follows: PCM, DPVWEYPLEFSWDTGWGDSS; scramble, WLSEAGPVVTVRALRGTGSW. To make MSN, firstly 1.0 g of CTAB (2.74 mmol) and 3.5 mL of NaOH solution (2 mmol) were dissolved in 520 mL of deionized water and heated to 80 °C, followed by the addition of 5.0 mL TEOS solution dropwise. The mixture solution was then stirred at 80 °C for 2 h. After 10 min centrifugation at 8700 g, the crude product was isolated and washed thoroughly with water and ethanol for three times. To remove CTAB, 0.7 g of the crude sample was dissolved in a mixture of ethanol (70 mL) and concentrated HCl (0.70 mL, 37.2%). Sixteen hours later, the organic solvent was rinsed using deionized water at least 3 times to obtain MSN. One gram of MSN was dissolved in 100 mL of anhydrous toluene via sonication, which was then incubated with 1 mL of APTS for 20 h. White particles were collected through centrifugation and sequentially washed three times with ethyl alcohol and deionized water, respectively. Following CTAB removal with 0.8 mL of HCl at 80 °C for 16 h, MSN-NH_2_ nanoparticles were obtained via lyophilization.

Subsequently, 100 mg of MSN-NH_2_ was dispersed in 20 mL of PBS with 0.03 mg of N-hydroxysuccinimide (NHS) and 0.3 mg of a 1-ethyl-3-(3-dimethylaminopropyl) carbodiimide (EDC) and stirred overnight at room temperature. PEG-MSN-NH_2_ was prepared with PEG co-incubation for 12 h via amidation. FITC-labeled scrambled peptide or FITC-labeled PCM in DMSO was incubated with PEG-MSN-NH_2_ for 28 h. The residue was extracted by centrifugation in 1000 pcf at room-temperature for 10 min three times and dried via lyophilization.

PCM-MSN@LA nanoparticles were prepared using 20 mg of PCM-MSNs dispersed in 10 mL of saturated LA solution and incubated in the dark for 20 h, and then washed for 3 times with deionized water followed by vacuum drying.

A total of 1 mg of PCM-MSN@LA was resuspended in 1 mL of PBS and stored at 4 °C before use.

### Characterization of PCM-MSN@LA

The features of PCM-MSN@LA nanoparticles including size, zeta, FTIR and morphology were assessed by DLS (Malven Zetasizer, Nano ZS90), infrared spectrometry (Nicolet, Avatar360) and transmission electron microscopy (TEM, FEI, TecnaiG2F20). Peptide conjugation and LA encapsulation were confirmed by thermal gravimetric analysis (Setaram, S60/51920). The mean size and zeta of nanoparticles were measured using DLS from day 0 to 28 for stability testing.

### ***L-arginine loading and L-arginine release using LIFU ***in vitro

Five milliliters of PCM-MSN@LA were dialyzed using a bag with 3500 Da MWCO (molecular weight cut-off) placed in an opaque centrifuge tube containing 20 mL of pH 7.35–7.45 or pH 7.00–7.30 HEPES buffer (50 mM HEPES, 2 mM CaCl_2_) on a shaker at 200 rpm min^−1^, 37 °C for 10 h. Every 36 min during 10 h, 100 μl of the buffer solution was withdrawn and mixed with 10% ninhydrin solution. The intensity of LA was recorded via an ultraviolet spectrophotometer (Shimadzu, UV2700) with a characteristic peak at 570 nm. For ultrasound-triggered LA release, LIFU (1.0 W/cm^2^-20% power-10 s per cycle, 10 cycles, 10 s intervals) was simultaneously performed on the bottom of the tube using a Sonitron 2000 V (Vepa Gene, Co., Ltd., Chiba, Japan). The absorbance intensities were recorded.

The function of LA molecules was indicated by the mass concentration acquired from loading and release profiles of LA according to a standard curve.

### Primary cardiomyocyte isolation

All animal experiments were approved by the Institutional Animal Care and Use Committee of Central South University (Changsha, China).

Cardiac tissue from 1-day-old C57/BL6 neonates were purchased from Hunan SJA Laboratory Animal Co., Ltd. (Hunan, China). To isolate cardiomyocytes, the heart was digested with 2.5 mg mL^−1^ trypsin(Aladdin Co., Ltd, Shanghai, China)and perfused with digestion buffer containing 2 mg mL^−1^ DNase I (Sigma, D7291). The cardiomyocytes were collected from the tissue piece suspension upon centrifugation and cultured in DMEM (HyClone, C11995500BT) containing 20% FBS (Biological Industries, 04-001-1ACS), 40 IU mL^−1^ penicillin (Sigma, P3032), 1.6 μg mL^−1^ VitB12.

(Sigma, V6629) and 0.1 mmol L^−1^ 5ˊ-bromo-2ˊ-deoxyuridine (Sigma, B5002) at 37 °C, 5% CO_2_, 21% O_2_ and 74% N_2_.

Male C57/BL6 mice aged 8 to 12 weeks (range 20–30 g) were purchased from Hunan SJA Laboratory Animal Co., Ltd. (Hunan, China), housed and maintained at room-temperature in an air-conditioned environment.

### The safety of PCM-MSN@LA

The changes in particle size and zeta from day 0 to 28 were measured using DLS.

#### Cell counting Kit 8 (CCK-8)

Cardiomyocytes were seeded in 96-well plates at a density of 1 × 10^4^ cells/well and cultured for 24 h. PCM-MSN@LA was added into the cells in triplicate at various concentrations of 0.05, 0.1, 0.2, 0.4, 0.6, 0.8, and 1 mg mL^−1^, respectively. After incubating for 24 h, the cells were collected and stained with a CCK-8 proliferation kit (Dojindo, CK04) The absorbance was measured at 450 nm.

#### Hematoxylin–eosin (HE) staining

The organs, including the lung, liver, spleen, kidney and heart, were isolated from C57/BL6 before and  24 h post 50 mg kg^−1^ PCM-MSN@LA injection. After 3–5 times of rinses with saline, some parts of each organ were fixed with 10% formalin and embedded in paraffin. Paraffin sections (≤ 5 μm) were stained with hematoxylin and eosin (HE) at 37 °C for 15 min. The slides were examined under a light microscope (Carl Zeiss AG, Oberkochen, Germany) at a magnification of 40 × 10.

### Evaluation of cell uptake and biodistribution of the targeted NO release system in the myocardium

To evaluate nanoparticle biodistribution, Cy5.5 fluorescent dye was used to replace L-arginine along with unstained PCM and scramble peptides. To demonstrate the specific binding ability of PCM to cardiomyocytes, cardiomyocytes treated with scrambled peptide-MSNs and HepG2 cells treated with PCM-MSNs were used as controls.

#### Cell colocalization staining

Both cardiomyocytes and HepG2 were seeded in 6-well plates at a density of 1 × 10^4^ cells per well. The cardiomyocytes were incubated with fresh DMEM containing 50 μg mL^−1^ PCM-MSN and scrambled peptide-MSNs, respectively, whereas HepG2 cells incubated with PCM-MSN only, for 1 h. After discarding the culture media, the cells were washed with PBS (pH 7.40) three times before 0.1% Triton X-100 permeabilization and 4% paraformaldehyde fixation. All cells were firstly stained with R-PE conjugated anti-sarcomeric alpha actinin (α-SA) (Abcam, 137346, 1:100) at 4 °C for overnight for cardiomyocyte-specific labeling [17]. After washing, the cells were counterstained with 4ˊ6-diamidino-2-phenylindole (DAPI) (Sigma, D9542) at room temperature for 15 min. Cardiomyocyte internalization was assessed using a confocal laser scanning microscope (CLSM, Olympus FluoView FV3000). The fluorescence intensity was graphed according to the white line of merged fluorescence images.

#### In vivo fluorescence imaging

Three groups of mice (4 mice each), MSN, PCM-MSN, and PCM-MSN + LIFU, were assessed for myocardial binding efficiency. After anesthetization with 1% pentobarbital sodium (50 mg kg^−1^), each type of nanoparticle solution was injected via the tail vein at a dose of 50 mg kg^−1^. The transducers were placed at the heart 1 min later. Ultrasound was transmitted at 1.0 W/cm^2^-20% duty power for 10 s per cycle, 10 cycles, 10 s intervals. Six hours later, the organs, including the heart, lungs, spleen, liver and kidneys, were isolated and rinsed with PBS for multiple times to wash away the blood. The distribution of fluorescence signal was visualized with in vivo imaging (PerkinElmer, IVIS lumina III). Heart cryosections (2 mm thickness) were prepared simultaneously to further assess nanoparticle localization in the cardiac. Quantitative analysis was performed using the Living Image 5.0 software.

### Measurement of intracellular NO release

Cardiomyocytes were cultured in DMEM with 10% FBS and 1% penicillin/streptomycin at a density of 1 × 10^6^ cells/well in a 6-well plate. The sepsis model was established by incubating the cells with 1 μg mL^−1^ LPS (Sigma, *Escherichia coli*, O111:B4), which was then added with PCM-MSN@LA at a concentration of 25, 50, 75 and 100 μg mL^−1^, respectively. Untreated cardiomyocytes with 25 or 50 μg mL^−1^ of PCM-MSN@LA were set up as control. Nitrite levels were measured with 1 mL of supernatant taken from each group at 2 h after treatment using a Griess assay kit (Beyotime Biotechnology, S0021, Haimen, China) on an ultraviolet spectrophotometer (Shimadzu, UV2700, Japan). For dynamic monitoring of NO generation in LPS-treated cardiomyocytes, the cells were incubated with 75 μg mL^−1^ PCM-MSN@LA and LIFU (1.0 W/cm^2^-20% duty power-10 s per cycle-10 cycles) was carried out at the bottom of the plate. The NO fluorescent probe DAF-FM DA (Beyotime Biotechnology, S0019) was added at different time points (pre, 5 min, 15 min, 30 min, 2 h and 6 h) and incubated with the cells at 37 °C for 20 min in darkness. Then the media was removed and the cells were washed 3 times with PBS. The fluorescence intensity in cardiomyocytes was measured by CLSM (Olympus FluoView FV3000) and quantified as nitrite levels.

### Cardiomyocytes viability and superoxide assessment.

In total, cardiomyocytes were prepared in 5 groups as Table 1 with various treatments. After 6 h, all cells were analyzed with a CCK-8 proliferation kit and the absorbance was measured at 450 nm.

**Table 1:** Viability and superoxide assessment

Group	Name	Treatment*
1	Control	None
2	LPS	LPS
3	PCM-MSN	PCM-MSN + LPS
4	PCM-MSN@LA	PCM-MSN@LA + LPS
5	PCM-MSN@LA + LIFU	PCM-MSN@LA + LPS, with LIFU irradiation

*LPS: 5 μg mL^−1^, PCM-MSN@LA: 75 μg mL^−1^

#### Ultrasound parameters

1.0 W/cm^2^–20% duty power, 10 cycles of 10 s irradiation with 10 s intervals

TUNEL staining (Beyotime Biotechnology, C1091) and DCFH-DA staining (Beyotime Biotechnology, S0033) were also conducted with cardiomyocytes seeded in 35 mm dishes with 0.17 mm thickness (Nest, N801001) at a density of 1 × 10^4^ cells per well after 24 h’ culture followed by treatments listed in Table 1 for 6 h. The cells were examined by laser confocal microscopy. The number of positive cells was calculated by Image-Pro Plus software version 6.0 (Media Cybernetics, Bethesda, MD).

### In vivo* myocardium-targeted NO production*

An endotoxic shock animal model was established with C57/BL6 mice. Lipopolysaccharide was dissolved in sterile PBS to a final concentration of 5 mg ml^−1^ (LPS). Pentobarbital sodium-anesthetized C57/BL6 mice were administered with 0.5 mL of PBS and LPS (10 mg kg^−1^ body weight), respectively, via intraperitoneal injection, followed by immediate subcutaneous injection of 1 mL saline (prewarmed to 37 °C) for fluid resuscitation. All measurements were conducted and samples were taken at 6 h after treatment unless otherwise indicated.

In total, 48 mice were divided into six groups as Table 2:

**Table 2:** In vivo NO production assessment

Group	Name	Treatment*
1	Control	Saline
2	LPS	LPS
3	PCM-MSN	PCM-MSN + LPS
4	MSN@LA	MSN@LA + LPS
5	PCM-MSN@LA	PCM-MSN@LA + LPS
6	PCM-MSN@LA + LIFU	PCM-MSN@LA + LPS, with LIFU irradiation

*PCM-MSN, MSN@LA and PCM-MSN@LA were all administrated at a dosage of 1 mg kg^−1^ for 2 min before LPS injection

LIFU was applied at 1 min after the last injection as follows: 1.0 W/cm^2^–20% duty power, 10 cycles of 10 s per cycle with 10 s intervals.

There were 36 mice divided additionally into six groups as listed in Table 2 for examining survival rates.

#### Measurement of blood cell markers

Levels of LDH, CK-MB and cTnI were measured with samples collected from the inferior vena cava and centrifuged at 589 g min^−1^ for 10 min, 4 °C. Plasma concentrations of LDH and CK-MB were measured using an automatic biochemical analyzer (Hitachi, 7600), whereas cTnI was measured by a Quantikine mouse ELISA kit (Cusabio, CSB-E08421).

#### Measurement of blood pressure

Systolic blood pressure (SBP) and diastolic blood pressure (DBP) were monitored using the P-600A automatic noninvasive blood pressure measurement system (TECHMAN, Chengdu, China). During the 6-h treatment, all mice had been placed on the receiver pad of a tail-cuff device with synchronized instrument clocks. All data have been recorded throughout the process.

#### Echocardiography

Echocardiography was performed at 6 h after CLP under light anesthesia with 2% isoflurane inhalation via an isoflurane delivery system. All images were obtained from an ultrasound scanner (FujiFilm, Visual Sonics Vevo 2100) coupled with a 30.0-MHz linear transducer. Left ventricular (LV) diameters at end-diastole and end-systole (LVEDD/LVESD) were measured from M-mode images in the short-axis view at the papillary muscle level. LV systolic function-fraction shortening (FS) and ejection fraction (EF) were calculated using Visual Sonics Measurement Software. Left ventricular volumes at end-diastole and end-systole (LV VOL d/LV VOL s) were evaluated using improved Simpson’s method by tracing the endocardium manually on the apical two Chamber view at end-diastole and end-systole for 3 cardiac cycles.

#### Histopathological assessment

Mouse heart tissue was fixed in 4% paraformaldehyde and paraffin-embedded before cutting into 5-μm sections for HE staining as mentioned above.

#### Electron microscopy assay

Mouse heart tissue was dissected into multiple slices with 1 mm thickness and fixed in 2.5% glutaraldehyde. After additional fixation in 1% osmium tetroxide (OsO4; Sinopharm Chemical Reagents Co., Ltd., Shanghai, China) for 1 h at room temperature, the sections underwent transmission electron microscopic examination (ZEISS 906, Germany).

#### Immunohistochemistry examination

To assess apoptosis, frozen mouse heart slides with 4-μm thickness were prepared in 15% sucrose at 4 °C. After blocking with avidin (Leagene Biotechnology Co., Ltd., Beijing, China) at room temperature for 20 min, the tissue samples were conducted with TUNEL staining following the manufacturer's instructions (KeyGEN BioTECH Co., Ltd., Jingsu, China). immune cells were stained using anti-rat Ly-6G antibody (Abcam, ab25377, 1:50) and goat anti-rat IgG secondary antibody (Life Technologies GmbH, Darmstadt, A10517, 1:200). Sections were washed 5 times and developed with a 3,3′-diaminobenzidine kit (DAB). All slides were counterstained with hematoxylin and examined by light microscopy (NIKON, ECLIPSE C1, Japan).

#### Immunofluorescence staining

Cellular reactive oxygen species were measured in mouse heart sections by dihydroethidium (DHE) labeling according to the manufacturer's instructions. After DHE (Sigma, D7008) staining for 30 min followed by DAPI staining for 5 min at 37 °C in darkness, the slides were analysed and imaged by fluorescence microscopy (Nikon Eclipse C1, Nikon, Japan).

#### Malondialdehyde (MDA) measurement

MDA is a stable metabolite of lipid peroxidation products. Heart samples were homogenized in 60 mL of PBS (pH 6). Tissue homogenate (0.1 mL) was mixed well with 0.2 mL MDA colorimetric reagent (Beyotime Biotechnology, S0131S). The mixture was incubated at 100 °C for 15 min in a water bath and then centrifuged for 10 min at room temperature. The absorbance at 532 nm was measured using a spectrophotometer (Cecil Instruments, Ltd., CE 9000, UK).

#### Western blot

Western blot was performed using mouse heart tissue homogenates with primary antibodies including iNOS (Abcam, 178945, 1:1000), eNOS (Abcam, 199956, 1:1000), NLRP3 (Adipogen, AG-20B-0014, 1:1000), NF-κB P65 (Cell Signaling Technology, 8242, 1:1000) and Cleaved-caspase3 (C-caspase3) (Cell Signaling Technology, 9964, 1:1000), and HRP-conjugated secondary antibodies. All images were developed via an ECL system (Amersham Biosciences, USA). Tubulin (Proteintech, 10094-1-AP, 1:5000) or GAPDH (Proteintech, 10494–1-AP 1:10000) antibody was used as an internal control.

#### Real-time PCR

Total RNA was extracted using RNAiso Plus (Takara), from each sample. cDNA was synthesized using the QuantScript RT Kit (TIANGEN) according to the manufacturer’s protocol. The levels of gene expression were amplified using PowerUp SYBR Green Master Mix'and analyzed by one-step RT-qPCR. The levels of gene expression were determined using the comparative Ct method and normalized to GAPDH. The oligonucleotide primers for IL-1β, IL-6, IL-18, TNF-α, and GAPDH are shown in Table 3.

**Table 3:** Primer sequences used in RT-qPCR experiments

Gene	Primer	Sequence
IL-1β	Forward	AAAAATGCCTCGTGCTGTCT
	Reverse	TCGTTGCTTGTCTCTCCTTG
IL-6	Forward	GGAGTTCCGTTTCTACCTGGA
	Reverse	TGGAAGTTGGGGTAGGAAGG
IL-18	Forward	TGACAAAAGAAACCCGCCTG
	Reverse	GGTCACAGCCAGTCCTCTTA
TNF-α	Forward	CGTCGTAGCAAACCACCAAG
	Reverse	AGCCTTGTCCCTTGAAGAGA
GAPDH	Forward	ACAGCAACAGGGTGGTGGAC
	Reverse	TTTGAGGGTGCAGCGAACTT

### Statistical analysis

Data were analysed using GraphPad Prism 5.0 software. Data are presented as the mean ± standard deviation (SD) in all of the experiments (n = 3). One-way ANOVA followed by Student’s t test was performed to determine the *P* values (95% confidence interval). Survival rates were evaluated with the log-rank test. The asterisks indicate statistical significance; unless otherwise specified, *P < 0.05, **P < 0.01.

## Supplementary Information

**Additional file 1**: **Fig. S1.** The X-ray diffraction pattern data shows that the nanoparticles exhibit diffraction peaks at (100), (110) and (200), indicating that the crystal is a hexagonal mesoporous array. **Fig. S2.** The TG methods shows that the PCM and L-arginine accounted for nearly 39.7 and 20.2% separately in mass. **Fig. S3.** The standard curve of L-arginine at various concentrations.

## Data Availability

All data generated or analyzed during this study are included in this published article.

## Abbreviations

NO: Nitric oxide
LPS: Lipopolysaccharide
MSN: Mesoporous silica nanoparticle
PCM: Primary cardiomyocytes by specific peptide
LIFU: Low-intensity focused ultrasound
NOS: Nitric oxide synthase
ONOO^−^: Peroxynitrite
eNOS: Endothelial NOS
nNOS: Neuronal NOS
iNOS: Inducible NOS
