# Supplementary figures and images for: Heart-targeted amelioration of sepsis-induced myocardial dysfunction by microenvironment responsive nitric oxide nanogenerators in situ
Source: J Nanobiotechnology. 2022 Jun 7;20:263. doi: 10.1186/s12951-022-01457-y (PMC9171488; doi:10.1186/s12951-022-01457-y)

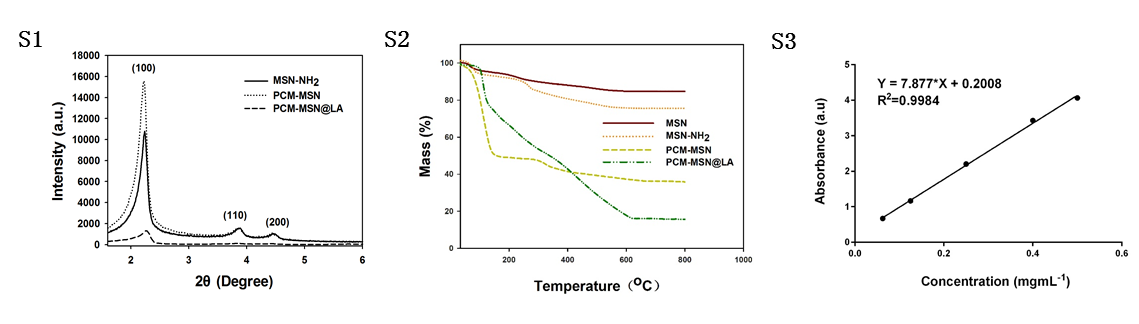

Supplement: Supplementary file 1 — Additional file 1: Fig. S1. The X-ray diffraction pattern data shows that the nanoparticles exhibit diffraction peaks at (100), (110) and (200), indicating that the crystal is a hexagonal mesoporous array. Fig. S2. The TG methods shows that the PCM and L-arginine accounted for nearly 39.7 and 20.2% separately in mass. Fig. S3. The standard curve of L-arginine at various concentrations. [file 12951_2022_1457_MOESM1_ESM.tif]
